# Evaluating the Risk of Tumors Diseases Based on Measurement of Urinary and Serumal Antioxidants Using the New Agar Diffusion Methods

**DOI:** 10.1155/2017/6578453

**Published:** 2017-03-28

**Authors:** Ying Zhou, Jing Chen, Zhen Wang, Hui Liu

**Affiliations:** ^1^College of Medical Laboratory, Dalian Medical University, Dalian 116044, China; ^2^School of Chemistry and Chemical Engineering, Shandong University, Jinan 250100, China

## Abstract

*Objectives*. To discuss the characteristics of the amount of urinary total antioxidants in tumor diseases and the possibility of utilizing the changing regulation of urinary antioxidants to diagnose tumor diseases.* Method*. Urine and serum specimens from 130 healthy people were used to investigate the variation of antioxidant capacity against age. Urine and serum specimens from 44 unselected patients with tumors and 44 healthy people with same age background were used to explore the significance of urinary antioxidant capacity in clinic to diagnose tumor diseases. Potassium permanganate agar method and iodine starch method were used to determine the amount of total antioxidants.* Results*. In healthy people, more antioxidants in urine were measured in older people, while the results were opposite in serum. More antioxidants were found in urine of tumor patients than in healthy people with same age-range.* Conclusions*. According to the results of 130 measurements, the amount of antioxidants in urine varies by age. By using agar methods to measure antioxidants, the effect of age is required to be considered. Antioxidants levels from tumor patients were significantly higher than healthy individuals in urine. The combination of urine and serum to determine total antioxidants can better diagnose tumor diseases based on iodine starch method, with area under the receiver operating characteristics curve at 0.787.

## 1. Introduction

Biological free radicals are general products of metabolism, mainly containing reactive oxygen species (ROS) and reactive nitrogen species (RNS). In common condition, the free radicals producing and removing are kept balanced, which play an important role in biological system. Once the balance is disturbed, free radicals in body can damage cells, tissues, and organs and further cause aging, cardiac diseases, brain diseases, and cancer [1, 2]. Thus, it is of great importance to know the total antioxidant capacity (TAC) of an organism to evaluate its free radicals producing/removing balance.

Nowadays, many researches are investigating the serum TAC [3], but few are focused on the urinary TAC. Human urine metabolome [4] has pointed out that thousands of compounds are detected in urine, including wide-ranged concentrations of urea, urobilinogen, inorganic salts, creatinine, ammonia, organic acids, and water-soluble toxins. As an important and easily accessible biological fluid, urine reflects the continuously changing environment of an organism [5]. Nevertheless, it has been proved that the level of urea [6], bilirubin [7, 8], and creatinine [9] in serum is associated with the TAC of an organism. So the measurement of antioxidants in urine is important; the variation of urinary metabolite profiling especially the TAC profiling may reveal specific disease.

In tumor patients, obvious oxidative stress is observed, indicating the balance between oxidants and antioxidants is broken up in each studied kind of tumors. In the development of tumors, there exists superfluous generation of ROS and RNS in organism [10]. ROS and RNS can cause DNA damage, protein damage, and lipid peroxidation [11–14]. ROS causes overexpression of Jun gene in lung cancer [15], while the increase of RNS causes protein damage in liver cancer [16]. Free radicals are involved in the initial, enhancement, and accumulation stage of tumor cells developing [17]. And the antioxidants in vivo can fight against with the surplus of free radicals. Low level of antioxidants bilirubin increases the risk of tumor related with smoking and alcohol [18, 19], while antioxidants uric acid is associated with DNA damage [20]. The amount of all the antioxidants in vivo reflects the capacity to react with the surplus of free radicals. However, it is unable to measure all the antioxidants because how many compounds are included in the antioxidants list is still unclear. Now, any proposed methods only cover a subset of the total antioxidants, which cannot truly reflect the redox state of an organism. A method that can measure the amount of total antioxidants in vivo is necessary.

Total antioxidants were previously investigated based on potassium permanganate agar and iodine starch agar method [21, 22]. In both methods, the diffusion area reflects TAC in an organism, and larger diffusion area represents better TAC. Both methods can better reflect the state of TAC than other methods such as ferric reducing antioxidant power assay [23, 24], cupric ion reducing antioxidant capacity assay [25, 26], and 2,2-diphenyl-1-picrylhydrazyl assay [27, 28].

In this work, potassium permanganate agar method and iodine starch agar method were used to determine the antioxidants in urine and serum, with the aim of applying antioxidant capacity to distinguish healthy people and tumor patients and to evaluate the risk of tumor diseases in clinic in a simpler way.

## 2. Materials and Method

### 2.1. The Variation of Total Antioxidants Measured in Urine and Serum in Healthy People

#### 2.1.1. Specimens

Urine specimens from 130 healthy people were collected from the first hospital affiliated with Dalian Medical University and the second hospital affiliated with Dalian Medical University. The urine specimens were divided into 13 groups equally for every five years; each group contained 5 males and 5 females; the ages of subjects ranged from 20 to 85 years. Serum specimens were also collected from the above. Urine and serum specimens were stored at −20°C after being collected and melted in room temperature before experiment.

#### 2.1.2. Method

The amount of total antioxidants in urine was determined by potassium permanganate agar method [21] and iodine starch agar method [22], respectively. Serum specimens were prepared and measured in the same way.

### 2.2. The Clinical Significance in Measuring the Amount of Total Antioxidants in Urine and Serum

#### 2.2.1. Specimens

Urine specimens were collected from the first hospital affiliated with Dalian Medical University and the second hospital affiliated with Dalian Medical University. Forty-four unselected patients with tumors were regarded as experimental group, including 19 males and 25 females, mean age at 57.84 ± 10.94 years. Forty-four healthy people were collected as control group, including 31 males and 13 females, mean age at 57.80 ± 11.45 years. The diagnoses of experimental group include gastric cancer, intestinal cancer, lung cancer, and breast cancer. Serum specimens were also collected from the above. Urine and serum specimens were stored at −20°C after being collected and restored in room temperature before experiment.

#### 2.2.2. Method

This section was the same as Section 2.1.2.

### 2.3. Statistical Analysis

Nonparametric correction was used to analyze the variation of antioxidant capacity in urine as well as serum. Nonparametric test was used to compare the results of urine diffusion. *T*-test was used to compare the results of serum diffusion. Receiver operating characteristics (ROC) curves were constructed to assess sensitivity, specificity, and respective areas under the curves (AUCs) with 95% confidence interval (CI). A value of *p* < 0.05 (two tailed) was considered significant. Statistical software package SPSS 13.0 was used to evaluate the results.

### 2.4. Ethical Approval

This article does not contain any studies with human participants or animals performed. The protocol has been approved by the Ethical Committee of Dalian Medical University.

## 3. Results and Discussion

In our previous publications, potassium permanganate agar method and iodine starch agar method were used to determine TAC in urine and serum [21, 22]. The diffusion area was dependent on the amount of antioxidants in urine and serum, in which larger diffusion area represents the fact that more antioxidants were measured. So, the amount of antioxidants reflects the antioxidant capacity in urine and serum; more antioxidants represent better antioxidant capacity. Both methods have good linearity and precision and can better reflect the state of TAC than reported methods in urine [22–26]. Neutral environment required in our methods is closer to physiological pH, high standard electrode potential of MnO_4_^−^/MnO_2_ can oxidize most antioxidants, and indicator starch-iodine is highly sensitive to judge the end point and the covering of liquid paraffin on the surface of the agar can exclude the interference of external O_2_.

### 3.1. The Variation of Total Antioxidants Measured in Healthy People by Potassium Permanganate Agar Method and Iodine Starch Agar Method

In Table 1, the amount of antioxidants was measured in both urine and serum of 130 healthy people. In urine, positive correction coefficients (*p* = 0.025 in potassium permanganate agar method and *p* = 0.016 in iodine starch agar method) between age and urine diffusion area represent the fact that more urinary antioxidants exist in older people than the younger age. Contrastingly, in serum, negative correction coefficients (*p* < 0.001 in potassium permanganate agar and *p* = 0.014 in iodine starch agar method) represent the fact that less antioxidants exist in sera from older people. Similar results were observed in both methods, which ensure the accuracy of the results that older people has more antioxidants in urine and less antioxidant in serum comparing with the younger ones.

The contrasting variations of antioxidants in urine and serum might be caused by the different antioxidant mechanisms. Future work is deserved to compare antioxidants profiling and fluid pathway in serum and urine. The question is whether the antioxidants in urine are derived from blood through glomerular filtration and tubular secretion.

The reason why we consider the age-background factor of healthy people is that, in previous reports, the serumal antioxidant capacity of the elderly will decline, but it is still unclear whether it is the same situation in urine. Thus, we measured the urinary antioxidants of healthy people to eliminate the age influence. Because there is age dependent trend observed between TAC and both urine and serum, the ages of tumor patients group and healthy group were designed to be matched to eliminate the influence.

### 3.2. The Total Antioxidants in Urine and Serum Were Measured in Unselected Patients with Tumors by Potassium Permanganate Agar Method and Iodine Starch Agar Method

Obvious oxidative stress is observed in patients with tumor. The balance between oxidants and antioxidants was broken up, regardless of the kinds of tumor. Here, clinical specimens of urine and serum were collected from patients with tumors without specifying the kind of tumors, which were labeled as “unselected patients with tumors” group.

In Table 2, because the urinary diffusion areas were not normally distributed in both methods, median diffusion areas were compared between the two groups. In potassium permanganate agar, the median diffusion area in tumor patients was 1.83 cm^2^, while it was 1.54 cm^2^ in healthy group; more antioxidants were measured in tumor patients, where *p* = 0.038; in iodine starch agar method, the median diffusion area in tumor patients was 1.54 cm^2^, while it was 1.13 cm^2^ in healthy group; more antioxidants were measured in tumor patients, where *p* = 0.016. While in serum the serum diffusion areas were normally distributed in both methods, average diffusion areas were compared between the two groups. In potassium permanganate agar, the average diffusion area in tumor patients was 2.02 ± 0.17 cm^2^, while it was 1.97 ± 0.17 cm^2^ in healthy group, because *p* = 0.158; no evidence showed that more antioxidants were measured in tumor patients; in iodine starch agar method, the average diffusion area in tumor patients was 3.20 ± 0.40 cm^2^, while it was 2.94 ± 0.46 cm^2^ in healthy group; more antioxidants were measured in tumor patients, where *p* = 0.005.

To ensure the accuracy of the results, only the results were significant in both methods; the amount of antioxidants was considered different between the two groups. Thus, more urinary antioxidants were observed in tumor patients, because the results were significant in both methods. However, there was no difference in the serum antioxidants between the two groups; the result was significant only in iodine starch method. From the data presented in Table 1, it can be seen in older healthy people that less serumal antioxidants were measured. This is opposite to the results presented in Table 2, where no more serumal antioxidants were measured in tumor patients. However, in urine, more antioxidants were measured in the both older group and tumor patients. Thus, it may indicate a fact that the occurrence of tumor diseases is related to not only aging but also other factors. Obviously, the antioxidant mechanism of aging and tumor diseases has its own characteristic, and the mechanism of tumor diseases is still to be explored. Thus, we can conclude that, in tumor diseases, the change of antioxidants in urine was more sensitive than that in serum. Compared with serum, urine is a better specimen to evaluate the risk of tumor diseases. Exploring the diagnosis value of measuring the total antioxidants in urine to evaluate the risk of tumor diseases was deserved.

### 3.3. Measuring the Urinary Total Antioxidants Can Improve the Diagnosis Efficiency of Tumor Diseases Based on the Measurement of Antioxidants in an Organism to Diagnose Tumor Diseases

**Figure 1:**
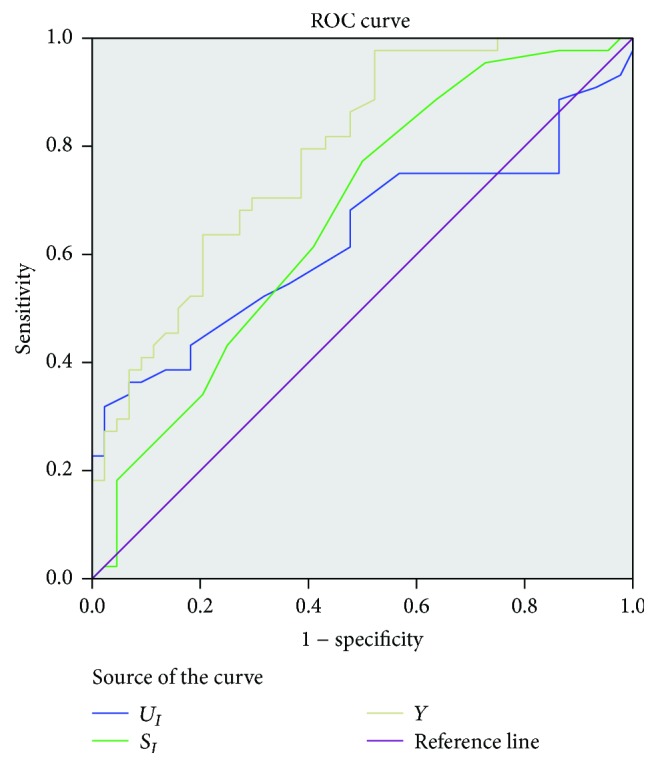
ROC curves for urine and serum in both potassium permanganate agar and iodine starch agar method.

In Table 4, four ROC curves for urine and serum in both methods were constructed, but all AUCs at either urine or serum were small. So the combination of them was in consideration. To assess the combined use of the measurement of urine diffusion area in potassium permanganate agar (*U*_Mn_), serum diffusion area in potassium permanganate agar (*S*_Mn_), urine diffusion area in iodine starch agar (*U*_*I*_), and serum diffusion area in iodine starch agar (*S*_*I*_), binary logistic regression was conducted. In Table 3, only *U*_*I*_ and *S*_*I*_ were significant in the regression model, where the *p* values were both 0.002. *U*_Mn_ and *S*_Mn_ were excluded; the logistical regression model was *Y* = 0.85*∗U*_*I*_ + 2.029*∗S*_*I*_ − 7.691. Thus, *U*_*I*_,  *S*_*I*_, and *Y* were chosen to build ROC, and it can be seen from Figure 1 that the AUC of *Y* was 0.787, which is larger than the use of *U*_*I*_ and *S*_*I*_ alone (Figure 1).

The reason why *U*_Mn_ and *S*_Mn_ were not selected in the regression model is that the sensitivity of potassium permanganate method is relatively lower than the iodine starch agar method; only few antioxidants that represent the differences between tumor diseases and healthy subjects were measured, but with the use of indicator starch, the sensitivity of iodine starch method was good; it can measure most antioxidants that represent the differences between tumor diseases and healthy subjects. And it also can be inferred that the electric potential of antioxidants in tumor patients was not high.

In all, the measurement of the urinary total antioxidants can improve the diagnosis efficiency of tumor diseases.

## 4. Conclusions

The antioxidant capacity of organism varies by age. More urinary antioxidants were measured in older people, while less antioxidants exist in serum of older people. The influence of age should be taken into consideration when discussing the TAC of an organism.

In patients with tumors, more antioxidants were found in urine with potassium permanganate agar method (*p* = 0.038) and iodine starch agar method (*p* = 0.016). More antioxidants were found in serum only in iodine starch agar method (*p* = 0.005). The change of the total amounts in urine was more sensitive than that in serum in tumor diseases.

The combined measurement of antioxidants in urine and serum could improve the diagnostic ability of tumor diseases, where AUC = 0.787. The measurement of total antioxidants in urine should be applied alone or in combination in clinic to evaluate the risk of tumor diseases.

## Figures and Tables

**Table 1:** The diffusion area (cm^2^) of urine and serum in healthy people in different groups (mean ± *S*).

Age groups	Urine		Serum	
	Potassium permanganate agar	Iodine starch agar	Potassium permanganate agar	Iodine starch agar
20~	3.35 ± 0.93	1.21 ± 0.25	2.52 ± 0.18	3.48 ± 0.51
25~	1.99 ± 1.42	1.19 ± 0.48	2.34 ± 0.15	3.15 ± 0.42
30~	2.24 ± 1.57	1.48 ± 0.51	2.41 ± 0.16	3.41 ± 0.39
35~	2.14 ± 0.96	1.22 ± 0.47	2.45 ± 0.16	3.36 ± 0.42
40~	2.15 ± 1.59	1.27 ± 0.32	2.39 ± 0.18	3.08 ± 0.30
45~	2.04 ± 1.17	1.10 ± 0.31	2.37 ± 0.21	3.37 ± 0.60
50~	2.33 ± 1.43	1.25 ± 0.44	2.37 ± 0.14	3.25 ± 0.47
55~	1.72 ± 0.98	1.38 ± 0.53	2.39 ± 0.13	3.29 ± 0.33
60~	3.10 ± 0.88	1.52 ± 0.50	2.26 ± 0.21	3.33 ± 0.48
65~	2.47 ± 1.29	1.57 ± 0.49	2.35 ± 0.19	3.44 ± 0.46
70~	2.28 ± 1.07	1.30 ± 0.51	2.28 ± 0.12	3.04 ± 0.27
75~	2.71 ± 1.73	1.22 ± 0.37	2.27 ± 0.33	3.24 ± 0.49
80~85	3.85 ± 1.27	1.47 ± 0.23	2.39 ± 0.22	3.03 ± 0.39

Correlation coefficient	0.139	0.150	−0.239	−0.152

*p*	0.025	0.016	<0.001	0.014

**Table 2:** Urine and serum diffusion area (cm^2^) of tumor patients and healthy people in the two methods.

Diffusion area (cm^2^)		Urine			Serum		
		Median	*Z*	*p*	Mean ± SD	*t*	*p*
KMnO_4_	Tumor patients	1.83	−2.076	0.038	2.02 ± 0.17	−1.424	0.158
	Healthy people	1.54			1.97 ± 0.17		

*I*_2_	Tumor patients	1.54	−2.399	0.016	3.20 ± 0.40	−2.867	0.005
	Healthy people	1.13			2.94 ± 0.46		

Because urinary diffusion areas in both methods were in nonnormal distribution, nonparametric test was used to compare the results in the two groups. While the serumal diffusion areas in both methods were in normal distribution, *t*-test was used to compare the results in the two groups. SD, standard deviation.

**Table 3:** Variables in binary logistic regression model.

Variables	B	SE	Wald	Sig.
*U*_*I*_	0.850	0.280	9.192	0.002
*S*_*I*_	2.029	0.651	9.702	0.002
Constant	−7.691	2.199	12.230	<0.001

*U*_*I*_, urine diffusion area in iodine starch agar; *S*_*I*_, serum diffusion area in iodine starch agar.

**Table 4:** Results for the measurement of total antioxidants in urine and serum in the diagnosis of tumor diseases.

Variable	AUC	Std. error	Asymptotic Sig.	95% CI
*U*_Mn_	0.626	0.060	0.043	0.509~0.742
*U*_*I*_	0.629	0.061	0.037	0.509~0.749
*S*_Mn_	0.503	0.064	0.963	0.377~0.629
*S*_*I*_	0.669	0.058	0.006	0.556~0.782

Logistical regression model	0.787	0.047	<0.001	0.694~0.880

*U*_Mn_, urine diffusion area in potassium permanganate agar; *U*_*I*_, urine diffusion area in iodine starch agar; *S*_Mn_, serum diffusion area in potassium permanganate agar; *S*_*I*_, serum diffusion area in iodine starch agar; AUC, area under curve; CI, confidence interval.
